# TRAF3 negatively regulates platelet activation and thrombosis

**DOI:** 10.1038/s41598-017-17189-1

**Published:** 2017-12-07

**Authors:** Rui Zhang, Guoying Zhang, Binggang Xiang, Xiaofeng Chen, Lijang Tang, Shaojun Shi, Yani Liu, Xun Ai, Ping Xie, Zhenyu Li

**Affiliations:** 1 Division of Cardiovascular Medicine, Department of Internal Medicine, College of Medicine, University of Kentucky, 741 South Limestone Street, Lexington, KY 40536 USA; 2 Department of Pharmacology, School of Basic Medicine, Tongji Medical College, Huazhong University of Science and Technology, Wuhan, 430030 China; 3 Taizhou Hospital, Wenzhou Medical University, Taizhou, 317000 China; 4 Department of Cardiology, Zhejiang Hospital, Hanzhou, 310013 China; 5 Department of Pharmacy, Union Hospital, Tongji Medical College, Huazhong University of Science and Technology, Wuhan, 43022 China; 6 Department of Physiology and Biophysics, Rush University, 1750 W Harrison St, Chicago, IL60612 USA; 7 Department of Cell Biology and Neuroscience, Rutgers University, 604 Allison Road, Piscataway, NJ 08854 USA

## Abstract

CD40 ligand (CD40L), a member of the tumor necrosis factor (TNF) superfamily, binds to CD40, leading to many effects depending on target cell type. Platelets express CD40L and are a major source of soluble CD40L. CD40L has been shown to potentiate platelet activation and thrombus formation, involving both CD40-dependent and -independent mechanisms. A family of proteins called TNF receptor associated factors (TRAFs) plays key roles in mediating CD40L-CD40 signaling. Platelets express several TRAFs. It has been shown that TRAF2 plays a role in CD40L-mediated platelet activation. Here we show that platelet also express TRAF3, which plays a negative role in regulating platelet activation. Thrombin- or collagen-induced platelet aggregation and secretion are increased in TRAF3 knockout mice. The expression levels of collagen receptor GPVI and integrin αIIbβ3 in platelets were not affected by deletion of TRAF3, suggesting that increased platelet activation in the TRAF3 knockout mice was not due to increased expression platelet receptors. Time to formation of thrombi in a FeCl_3_-induced thrombosis model was significantly shortened in the TRAF3 knockout mice. However, mouse tail-bleeding times were not affected by deletion of TRAF3. Thus, TRAF3 plays a negative role in platelet activation and in thrombus formation *in vivo*.

## Introduction

CD40, a phosphorylated membrane glycoprotein, belongs to TNF receptor superfamily^1–3^. CD40 plays an important role in adaptive immunity and inflammation. CD40L is primarily expressed on activated T cells^4^. Platelets express both CD40 and CD40L^5–8^. CD40L potentiates platelet aggregation *in vitro* ^7,9^. Recombinant sCD40L has been shown to specifically bind to purified integrin αIIbβ3 and activate platelets in a β3-dependent manner^10^. However, increase in αIIbβ3 activation caused by sCD40L was abrogated by both CD40 and CD40L blocking antibodies^7^. These data indicate that CD40L-potentiated platelet activation also requires the CD40L-CD40 interaction. Besides its role in thrombosis, platelet-derived CD40L plays a role in the development of atherosclerosis through inhibiting vascular endothelial growth factor-induced endothelial cell migration^11^.

CD40 signaling depends on the family of cytoplasmic adapter proteins known as TNF receptor-associated factors (TRAFs)^12,13^. TRAFs share a relatively conserved secondary structure and mediate signaling of the transmembrane TNF receptors and CD40 as well. TRAFs consist of seven known members, TRAF1~7. Unlike other TRAF members such as TRAF2, TRAF5, and TRAF6 that mediate the CD40-induced activation of the transcription factors, TRAF3 blocks CD40-induced signaling^14–17^. Platelets express several TRAF members, including TRAF1, 2, and 6^18^. It has been shown that sCD40L stimulation induces the association of TRAF2 with platelet CD40 and sCD40L potentiates platelet activation through a TRAF2/Rac1/p38 MAPK signaling pathway^18^. TRAF3 is expressed in a variety of immune cells and mediates signaling of the TNF receptor superfamily, including TLRs, NOD-like receptors, and RIG-I–like receptors^19,20^. In this study, we show that TRAF3 is highly expressed in human and mouse platelets and plays a negative role in regulating platelet activation. Furthermore, our data indicate that TRAF3 plays an important role in thrombus formation *in vivo*.

## Results

### TRAF3 is expressed in platelets

**Figure 1:**
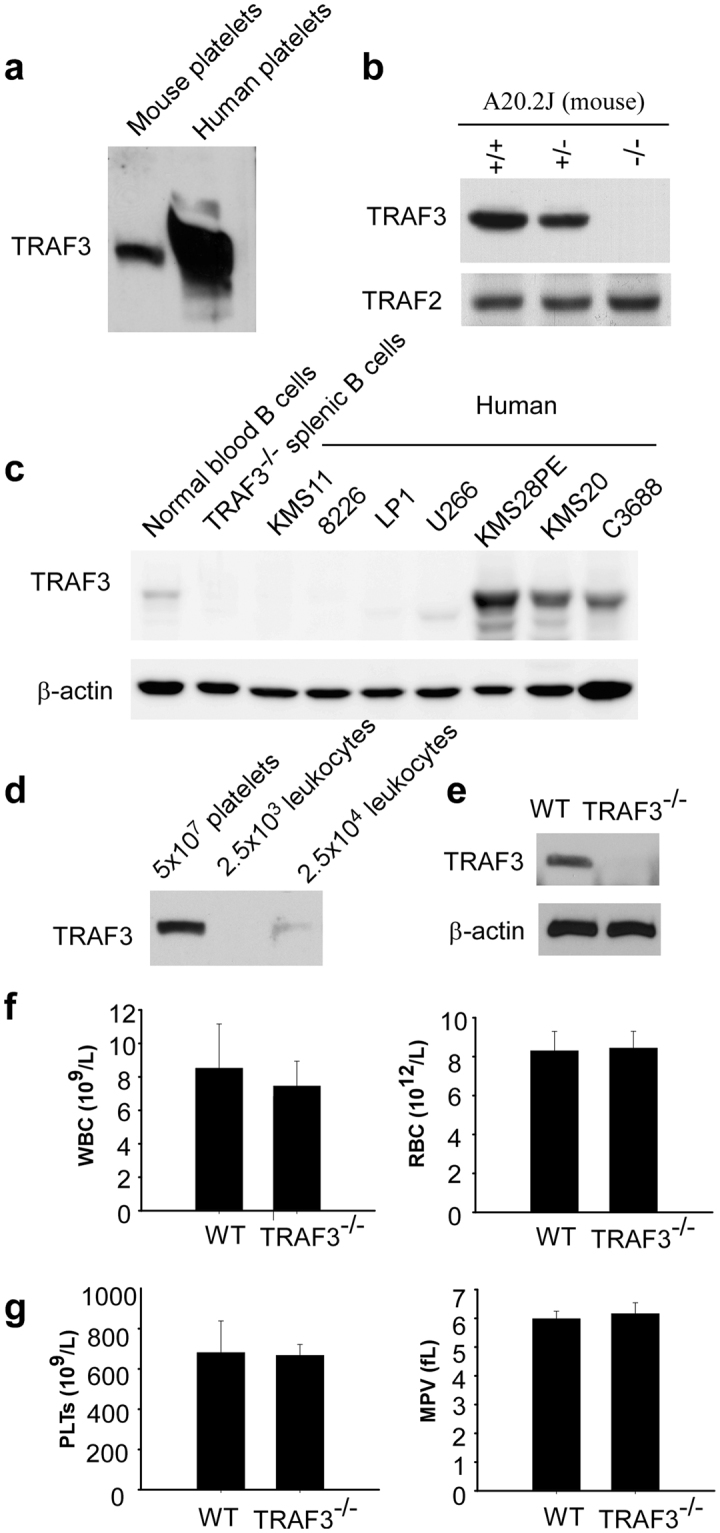
TRAF3 is expressed in platelets. (**a**) TRAF3 in human and mouse platelet lysates (5 × 10^7^ platelets/lane) was detected by Western blot with polyclonal anti-TRAF3 antibody. (**b**) Validation of the TRAF3 antibody using TRAF3^−/−^ mouse B cell line A20.2 J lysates in Western blot. Protein samples were immunoblotted for TRAF3, stripped, and reimmunoblotted for TRAF2. (**c**) Validation of the TRAF3 antibody using TRAF3-deficient human patient-derived multiple myeloma cell lines in Western blot. Human cell lines examined include two cell lines with TRAF3 bi-allelic deletions (KMS11 and 8226), two cell lines with TRAF3 frameshift mutations (LP1 and U266), two cell lines with cIAP1/2 bi-allelic deletions and wild type TRAF3 (KMS28PE and KMS20), and an EBV-transformed B lymphoblastoid cell line with wild type TRAF3 (C3688). Protein samples were immunoblotted for TRAF3, stripped, and reimmunoblotted for actin. (**d**) TRAF3 in lysate of mouse platelets (5 × 10^7^) and lysates of leukocytes (2.5 × 10^3^ and 2.5 × 10^4^, respectively) was detected by Western blot with polyclonal anti-TRAF3 antibody. (**e**) TRAF3 in platelet lysates of TRAF3^+/+^ and TRAF3^−/−^ mice was detected by Western blot. All the experiments were repeated at least three times. (**f**) White blood cell counts and red blood counts in TRAF3^+/+^ and TRAF3^−/−^ mice (n = 4 for each group). (**g**) Platelet counts and size of TRAF3^+/+^ and TRAF3^−/−^ mice (n = 4 for each group).

To determine whether TRAF3 is expressed in platelets, we examined the presence of TRAF3 in platelet lysates using Western blot with a polyclonal antibody against TRAF3. Our results indicate that TRAF3 is present in both human and mouse platelets (Fig. 1a). TRAF3 was detected in wild-type mouse B cell lysate but not in the lysate of B cells from the TRAF3 deficient mice (Fig. 1b). These data suggest that this antibody specifically recognizes TRAF3. To further validate the specificity of the antibody, we detected TRAF3 in the lysates of human cell lines KMS11, 8226, LP1, and U266 that are deficient of TRAF3. TRAF3 was undetectable in the lysates of these cell lines. In contrast, this antibody detected TRAF3 in the lysates of the cell lines KMS28PE, KMS20, and C3688 that express TRFA3 (Fig. 1c). Because TRAF3 is expressed in leukocytes, to verify that the detected TRAF3 in platelet lysates was not from contaminated white blood cells, we measured contamination levels of leukocytes in platelets using a HEMAVET HV950FS multispecies hematology analyzer. In our platelet preparation, leukocyte contamination level is 3.82 ± 2.47 × 10^3^ in 5 × 10^7^ platelets (n = 8). We then compared TRAF3 expression levels between platelets and leukocytes by Western blot. TRAF3 was undetectable in the lysate of leukocytes at the contamination level (Fig. 1d). The amount of TRAF3 in the leukocyte lysate at 10 time contamination level was much lower than that of platelets. Therefore, it is unlikely that the detected TRAF3 in platelet lysates was from contaminated leukocytes.

There are no commercial TRAF3 inhibitors. To investigate the role of TRAF3 in platelet function and thrombosis, we generated megakaryocyte- and platelet-specific knockout mice through breeding of the TRAF3^fl/fl^ mice with *Pf4*-Cre recombinase transgenic mice. Immunoblotting demonstrated complete absence of TRAF3 in the platelets from TRAF3^fl/fl^/Cre^+^ (TRAF3 knockout) mice (Fig. 1e). In contrast, TRAF3 is expressed in the platelets from the wild-type littermates (TRAF3^fl/fl^). Mice lacking TRAF3 in platelets appeared to develop normally and had normal white blood cell count and red blood cell count (Fig. 1f). Platelet counts and platelet size were not different between TRAF3 knockout mice and wild-type littermates (Fig. 1g).

### Deficiency of TRAF3 potentiates platelet activation

**Figure 2:**
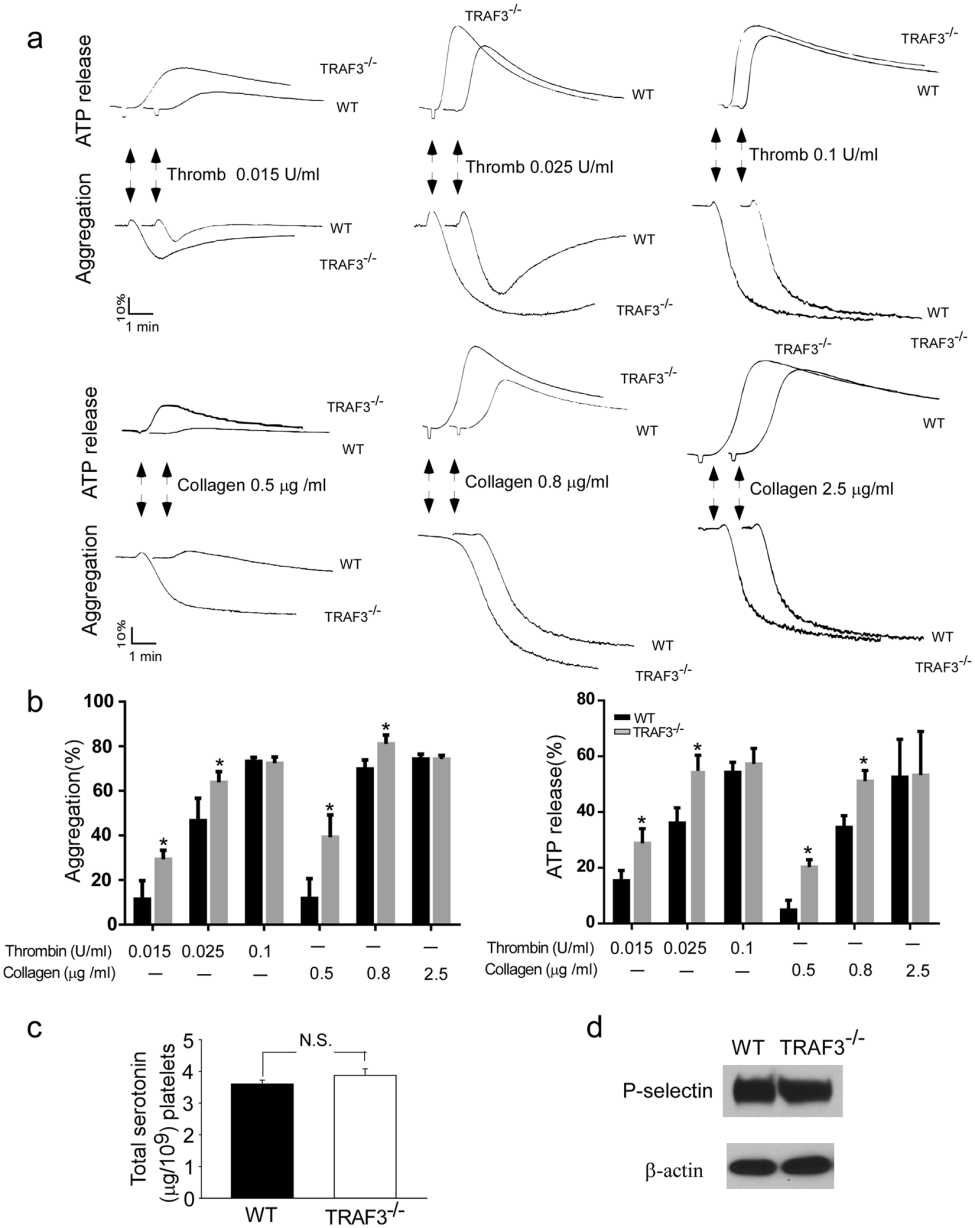
Platelet secretion and aggregation are defective in the TRAF3 knockout mice. (**a**) Washed platelets from TRAF3 knockout mice (TRAF3^−/−^) or wild-type littermates (TRAF3^+/+^) were stimulated with various concentrations of thrombin or collagen and simultaneously recorded for ATP secretion and aggregation. The aggregation and ATP release traces are representatives of at least three different experiments. (**b**) Aggregation and ATP secretion results in the experiments described in A were quantitated. (**c**) Total serotonin in 1 × 10^9^ TRAF3^+/+^ and TRAF3^−/−^ platelets from four mice with each genotype was measured by O-phthalaldehyde assay as described under “Experimental Procedures” (n = 4). (**d**) Detection of P-selectin in platelet lysates from TRAF3^+/+^ and TRAF3^−/−^ mice by Western blot (n = 4).

To investigate the role of TRAF3 in platelet activation, we compared platelet aggregation and ATP release in response to agonists between TRAF3 knockout mice and wild-type littermates. Platelet aggregation and ATP secretion elicited by low-dose agonists were increased in TRAF3^−/−^ platelets (Fig. 2a,b). At high concentrations, thrombin and collagen elicited similar levels of platelet aggregation and ATP release in TRAF3^−/−^ and TRAF3^+/+^ platelets.

To exclude a possibility that increased secretion in TRAF3^−/−^ was due to increased granule contents, we compared the total amount of serotonin and P-selectin expression, as markers for dense and α granules respectively, between TRAF3^−/−^ and TRAF3^+/+^ platelets. The amount of serotonin and the expression level of P-selectin were similar between TRAF3^−/−^ and TRAF3^+/+^ platelets (Fig. 2c,d).

### Potentiation of platelet activation by TRAF3 deficiency does not require p38 MAPK and Akt pathways

**Figure 3:**
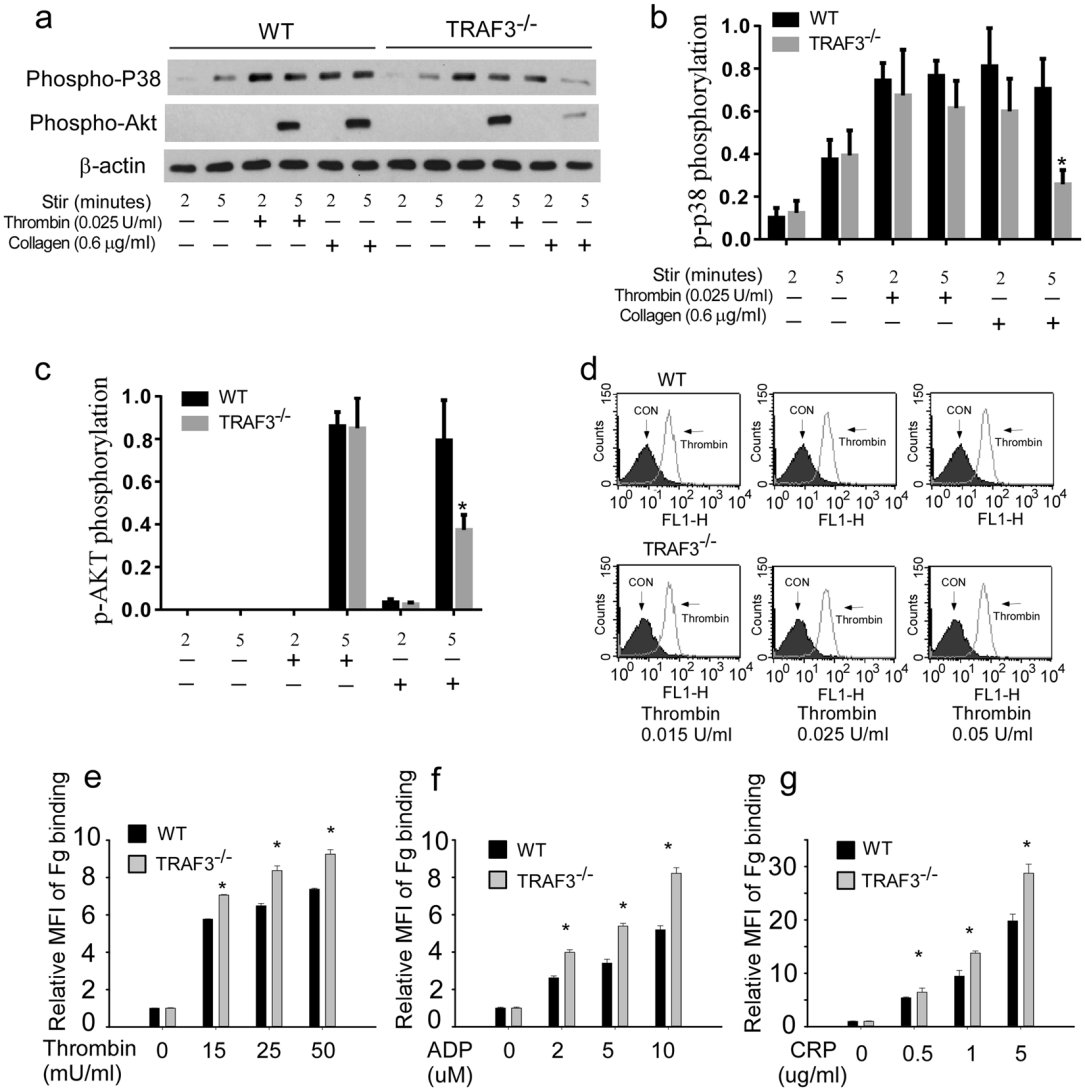
Akt and p38 MAPK phosphorylation and fibrinogen binding in response to thrombin in TRAF3^+/+^ and TRAF3^−/−^ platelets. (**a–c**) Washed platelets from TRAF3^+/+^ and TRAF3^−/−^ mice were incubated with increasing concentrations of thrombin or collagen at 37 °C in a platelet aggregometer for 2 or 5 min, and solubilized with SDS-PAGE sample buffer. Phosphorylation of Akt and p38 MAPK was detected by Western blotting with rabbit monoclonal antibodies specifically recognizing the phosphorylated Akt residue Ser473 or phosphorylated p38 residues Thr180/Tyr182. Statistical data of densitometric analysis from at least four experiments were shown in (**b**) and (**c**). (**d** and **e**) Washed platelets from TRAF3^+/+^ and TRAF3^−/−^ mice (3 × 10^8^/ml) were incubated with Oregon Green-labeled fibrinogen in the presence of various concentrations of thrombin at 22 °C for 30 min. Platelets were also incubated with Oregon Green-labeled fibrinogen in the absence of thrombin at 22 °C for 30 min as a control. Fibrinogen binding to platelets was analyzed by flow cytometry. Quantitative results were expressed as fibrinogen binding indices (geometric mean (MFI) mean of fluorescence intensity of stimulated platelets/ geometric mean of fluorescence intensity of unstimulated platelets; geometric mean ± SD; n = 3; *P < 0.05). (**f** and **g**) Fibrinogen binding to platelets induced by ADP and CRP was analyzed by flow cytometry (geometric mean of geometric fluorescence intensity of stimulated platelets/mean of fluorescence intensity of unstimulated platelets; mean ± SD; n = 4; *P < 0.05).

CD40L-mediated platelet activation involves several signaling pathways, including the PI3Kβ/Akt pathway and the p38 mitogen-activated protein kinase (MAPK) signaling pathway^7,21^, which are known to play important roles in mediating platelet activation^22^. Therefore, we examined whether TRAF3 negatively regulates platelet activation by inhibiting the activation of Akt and p38 MAPK in response to agonists. Akt and p38 MAPK phosphorylation in response to thrombin was similar between TRAF3^+/+^ and TRAF3^−/−^ platelets (Fig. 3a–c). Collagen-induced Akt and p38 phosphorylation was even reduced in the TRAF3^−/−^ platelets (Fig. 3a–c). Thus, it is unlikely that TRAF3 inhibits platelet activation by affecting those pathways. Previous studies have reported that CD40L can activate integrin αIIbβ3. Therefore, we investigated whether TRAF3 potentiates platelet activation through enhancing integrin αIIbβ3 activation. Although the percentage of fibrinogen-positive platelets is not different between WT and TRAF3 KO in the manuscript, total amount of fibrinogen bound to platelets was increased in the TRAF3 knockout mice in response to thrombin (Fig. 3d,e). Similarly, ADP- or CRP-induced fibrinogen binding was also increased by TRAF3 deficiency (Fig. 3f,g, and Supplementary Information file (Fig. S1)).

### CD40L potentiated platelet activation in both wild type mice and TRAF3 knockout mice

**Figure 4:**
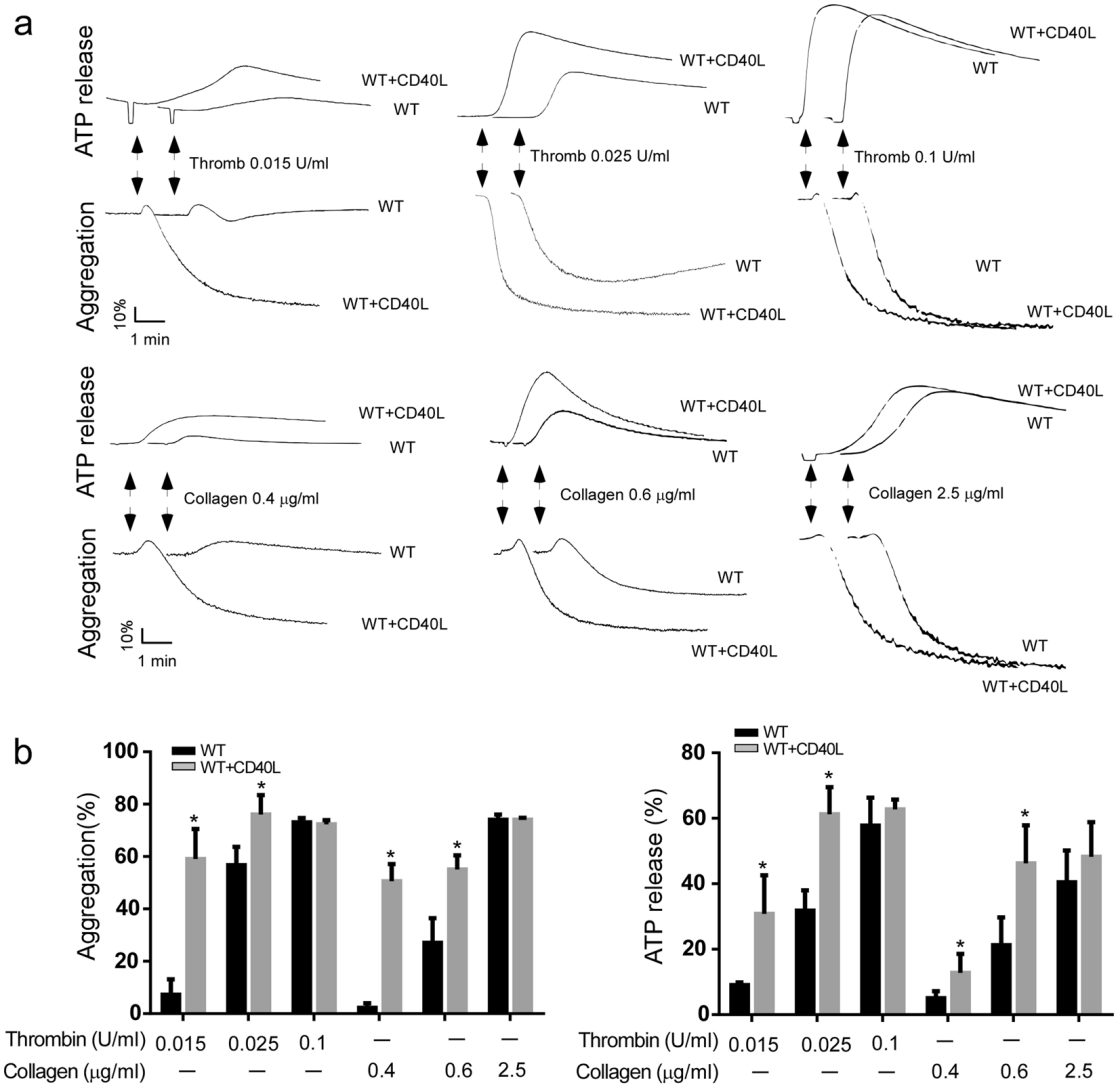
CD40L potentiates platelet aggregation and secretion in TRAF3^+/+^ mice. (**a** and **b**) Washed platelets (3 × 10^8^/ml) from TRAF3^+/+^ mice were exposed to thrombin or collagen in the presence or absence of CD40L to induce platelet aggregation and ATP release. The aggregation and ATP release traces are representatives of at least three different experiments. Aggregation and ATP secretion results in the experiments described in (**a**) were quantitated and shown in (**b**) (n = 3, *p < 0.05).

**Figure 5:**
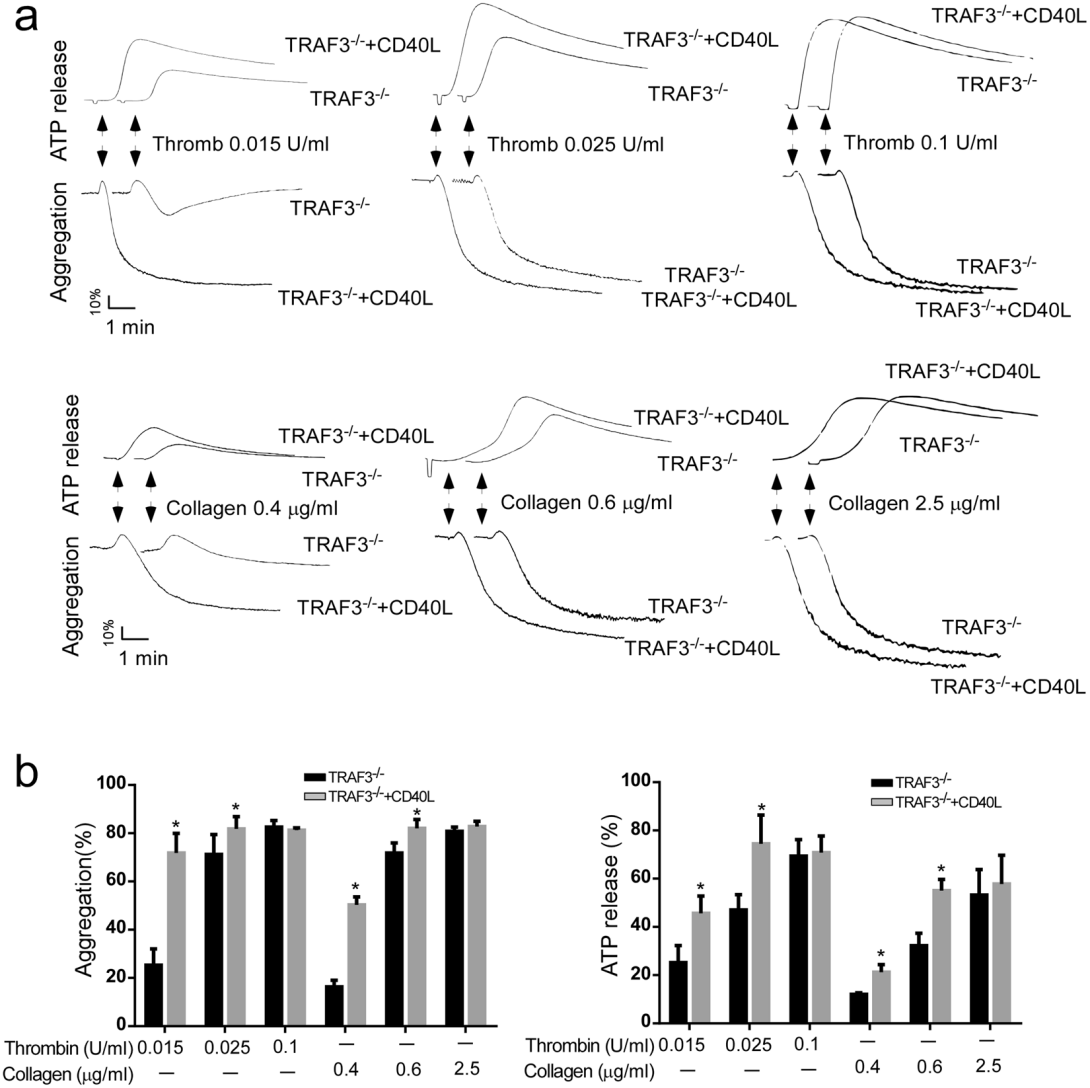
CD40L potentiates platelet aggregation and secretion in TRAF3^−/−^ mice. (**a** and **b**) Washed platelets (3 × 10^8^/ml) from TRAF3^+/+^ mice were exposed to thrombin or collagen in the presence or absence of CD40L to induce platelet aggregation and ATP release. The aggregation and ATP release traces are representatives of at least three different experiments. Aggregation and ATP secretion results in the experiments described in (**a**) were quantitated and shown in (**b**) (n = 3, *p < 0.05).

Previously studies reported that addition of exogenous CD40L potentiated platelet secretion and activation. To determine whether TRAF3 is involved in CD40L-mediated platelet activation, washed platelets from wild-type and TRAF3 knockout (TRAF3^−/−^) mice were exposed to low-dose platelet agonists in the presence of CD40L. As expected, platelet aggregation and ATP release in response to low-dose thrombin or collagen, were increased in wild-type mice by addition of CD40L (Fig. 4). CD40L also increased platelet aggregation and ATP secretion in TRAF3 knockout mice (Fig. 5). These data suggest that TRAF3 is not required for CD40L-potentiated platelet activation.

### Expression of integrin or GPVI on platelets is not affected by deletion of TRAF3

To exclude a possibility that increased platelet activation in the platelets lacking TRAF3 is due to increased expression levels of receptors, we compared expression of integrin αIIbβ3 and GPVI in platelets between the TRAF3 knockout mice and wild-type littermates using flow cytometry with FITC-labeled monoclonal antibodies against β3 or GPVI. The expression levels of β3 and GPVI are similar between TRAF3 knockout and wild-type littermates (Supplementary Information file (Fig. S2a, b)). These results were confirmed by Western blot with antibodies against β3 or GPVI (Supplementary Information file (Fig. S2c, d)).

### Increased platelet activation by lack of TRAF3 is not due to enhanced expression of other TRAFs

TRAF2 has been shown to play a role in mediating CD40L-dependent platelet activation. To assess a possibility that increased platelet activation by TRAF3 knockout is due to enhanced expression of TRAF2, we compared the expression level of TRAF2 in platelets between TRAF3 knockout mice and wild-type littermates. The expression level of TRAF2 in platelets between TRAF3 knockout mice and wild-type littermates was similar (Supplementary Information file (Fig. S2d, e). In addition, TRAF3 deficiency did not affect expression of other TRAFs, including TRAF 5, 6, and 7 and the expression of CD40 (Supplementary Information file (Fig. S2f)).

### The role of TRAF3 in thrombosis and hemostasis *in vivo*

**Figure 6:**
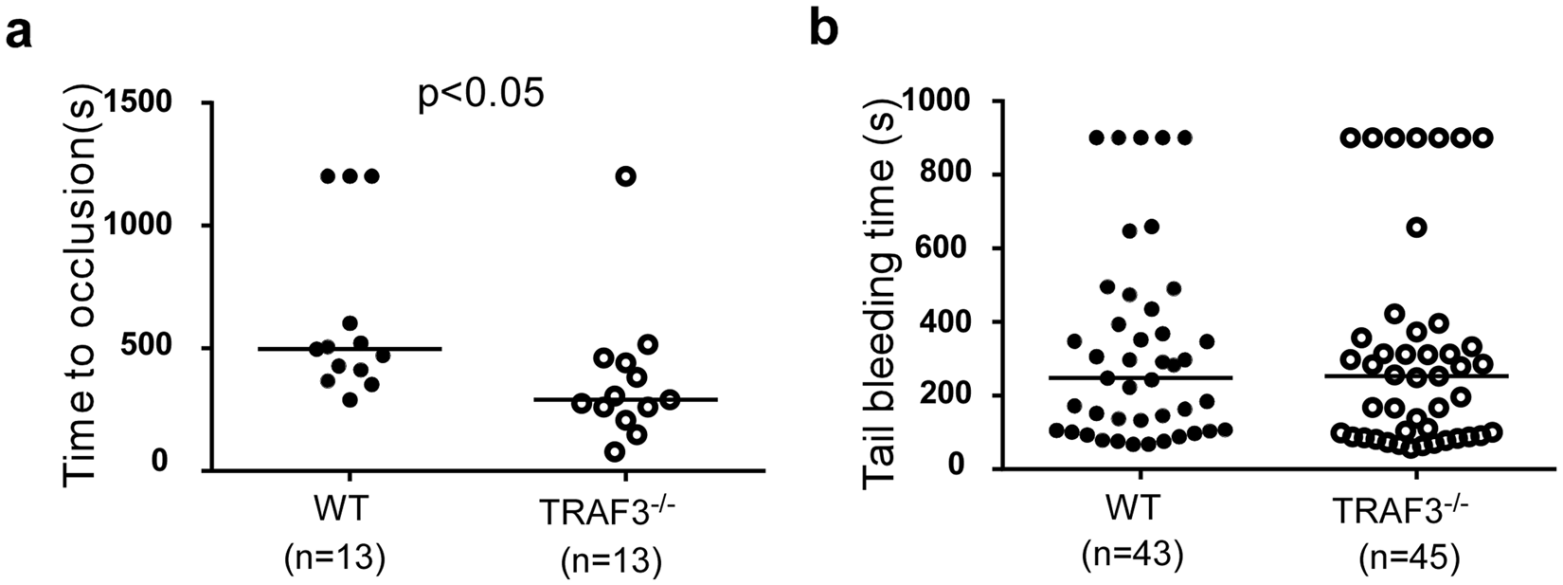
TRAF3 knockout potentiated thrombosis but did not affect hemostasis. (**a**) FeCl_3_-induced carotid artery injury was performed and time to occlusive thrombosis recorded as described under *Experimental Procedures*. The occlusion time of each mouse is shown as circles. The bars represent the median occlusion time (282.5 s for TRAF3^−/−^, and 483.5 s for TRAF3^+/+^, n = 13 for each group). The difference in occlusion time between TRAF3^+/+^ and TRAF3^−/−^ mice is statistically significant (p = 0.010 by Nonparametric Mann-Whitney test). (**b**) Bleeding time tests were performed blind to genotype in TRAF3^+/+^ (8–10 weeks old, n = 43) and TRAF3^−/−^ littermates (n = 45) generated from mating TRAF3^+/−^ mice. The solid triangles represent the bleeding time of a single mouse. The bars represent the median bleeding time of the group (251.0 s for TRAF3^−/−^, and 245.5 s for TRAF3^+/+^). The difference in bleeding time between TRAF3^+/+^ and TRAF3^−/−^ mice is not statistically significant (p > 0.1 by Nonparametric Mann-Whitney test).

Platelet activation is critical in the process of thrombosis and hemostasis. Thus, we further investigated whether TRAF3 knockout affected the *in vivo* thrombus formation using the FeCl_3_-injured carotid artery thrombosis model. The time to the formation of stable thrombus in TRAF3^−/−^ mice (median, 282.5 seconds, n = 13) is significantly shortened, compared to wild type mice (median, 483.5 seconds, n = 13) (p = 0.0105) (Fig. 6a). Tail-bleeding time analysis indicated that the median bleeding time was 245.5 seconds (n = 43) in wild-type mice and the median bleeding time of TRAF3 knockout mice was 251.0 seconds (n = 45, p > 0.1) (Fig. 6b). Thus, TRAF3 plays an important role in the regulation of thrombus formation but does not appear to affect hemostasis *in vivo*.

## Discussion

Our data reveal the expression of TRAF3 in platelets. Importantly, we present a novel finding that TRAF3 plays a negative role in regulating agonist-induced platelet activation as well as in thrombosis *in vivo*.

Although it has been known for many years that CD40L is an important positive regulator of platelet activation and thrombosis, the mechanism by which CD40L regulates platelet activation is not completely understood. Early studies demonstrated CD40L as a ligand of integrin αIIbβ3^10^. CD40L appears to bind directly to the integrin β3 subunit through the KGD sequence and is necessary for stability of arterial thrombi^10^. Recombinant soluble CD40L promotes integrin activation and platelet aggregation under high shear rates^10^. CD40L-dependent integrin activation can be abrogated by blocking antibodies against either CD40L or CD40, indicating the CD40L-CD40 axis is also required for CD40L-promoted integrin activation^7^. These findings are consistent with another study showing that sCD40L enhances platelet activation and aggregation in wild-type but not in the CD40^−/−^ knockout mice^21^.

We found that TRAF3 is highly expressed in human and mouse platelets. These findings appear to contradict with proteomics studies^23,24^, in which TRAF3 was not detected in human and mouse platelet samples. However, there are many reasons for failure to detect TRAF3 by proteomics. For example, the method of sample pretreatment may affect mass spectrometry for protein detection. In addition, the parameters of mass spectrometry may also affect the degree of ionization of the sample, such as ion spray voltage, declustering potential, collision energy, etc. TRAF1 is highly expressed in platelets^18^ but was not detected by the proteomics studies^23,24^. Our findings appear also to contradict with a previous study, which showed that TRAF3 was minimal in platelets^7^. The discrepancy between the previous study and our study may be due to different antibodies against TRAF3 used, because we could not detect TRAF3 in platelets or other cells by Western blot with the same antibody used in the previous study^7^. To validate the antibody that used in Fig. 1, we detected TRAF3 in mouse and human B cell lysates as positive controls and human samples deficient of TRAF3 as negative controls. Our data indicate that this antibody can detect TRAF3, because it recognizes a band with expected molecular weight in both mouse and human B cells samples by Western blot, but the band is absent in the samples that are deficient of TRAF3 (Fig. 1b,c).

In immune cells, signaling of CD40L-CD40 axis is mediated by TRAFs. Platelets express TRAF1, 2, and 6^7^. sCD40L stimulation of platelets induces the association of TRAF2 with CD40^7^, suggesting that TRAF2 may be involved in CD40L-mediated platelet activation. Our data indicate that platelet aggregation and secretion in response to low-dose agonist are increased in the TRAF3 knockout mice, suggesting that TRAF3 is a negative regulator of platelet activation. This conclusion is consistent with the findings that overexpression of TRAF3 prevents CD40-signaling in endothelial cells^25^. Accordingly, times to formation of occlusive thrombi are shortened in the TRAF3 knockout mice. It is unknown whether TRAF3 regulates platelet activation through a CD40L-CD40-dependent mechanism or a CD40L-CD40-independent mechanism. Although platelet aggregation in the absence of exogenous CD40L is increased, these data do not exclude the possibility that potentiation of platelet aggregation by TRAF3 knockout might still be CD40L-CD40, because platelet can secrete endogenous CD40L upon activation. This possibility could be addressed using CD40L and TRAF3 double knockout mice in future studies.

The mechanism by which TRAF3 regulates platelet activation is under investigation. Although CD40L-mediated platelet activation involves PI3Kβ/Akt pathway and the p38 mitogen-activated protein kinase (MAPK) signaling pathway^7,21^, Akt and p38 phosphorylation was not enhanced in the TRAF3 deficient platelets. These data suggest that inhibition of platelet activation by TRAF3 appears not to involve those pathways. Using the FeCl_3_-induced carotid artery thrombosis model, we investigated the role of TRAF3 in thrombus formation. It has been shown that FeCl_3_-induced thrombosis may be not only due to the injury of vessel wall, but also due to its effects beyond the vessel wall, including charge-dependent aggregation, effects of FeCl_3_ on blood cells and plasma proteins, and tissue factor generation^26–30^. Although the mechanism by which FeCl_3_ induces thrombosis has not been fully understood, numerous studies indicate that FeCl_3_-induced thrombosis is an effective and reliable assay for platelet function^10,26,28,31–35^. Consistent with the enhanced reactivity of the TRAF3 deficient platelets, deficiency of TRAF3 potentiated thrombosis in a FeCl_3_-induced carotid artery thrombosis model. Interestingly, although TRAF3 deficiency has a “modest” effect on aggregation in the *in vitro* assays, TRAF3 knockout mice appears to have a significantly phenotype in the *in vivo* thrombosis model. The phenomenon has been reported previously. It has been observed that some knockout mice lacking a certain protein have defects in platelet aggregation and integrin activation only in response to low doses of agonists, but show significant defects in the tail transection model and thrombosis in the FeCl_3_-induced carotid injury model^36–38^. It appears that the nature of thrombosis needs a robust response of platelets, which involves multiple receptors and signaling molecules. Therefore, a “small” effect on the *in vitro* platelet function assays may significantly affect the *in vivo* thrombosis. Another possibility for this phenotype is that the sensitivity of the *in vivo* assay is much higher than the *in vitro* assays. In addition, shear stress, vessel contractility, and the interaction of platelets with the endothelium may also contribute to a different magnitude of effect when comparing *in vitro* with *in vivo* data.

## Materials and Methods

### Materials

Luciferin/luciferase reagent and collagen were purchased from Chronolog, Havertown, PA. Human α-thrombin was from Enzyme Research Laboratories, South Bend, IN. CD40L was purchased from eBioscience (San Diego, CA, USA). Rabbit monoclonal antibodies against phosphorylated Ser^473^ residue of Akt and against phosphorylated Thr^180^/Tyr^182^ residues of p38 MAPK were from Cell Signaling Technology (Beverly, MA, USA). A rabbit polyclonal antibody against TRAF3 was purchased from Santa Cruz Biotechnology Inc. (Santa Cruz, CA, USA). A rabbit polyclonal antibody against TRAF2 was purchased from NeoBiolab (Woburn, MA, USA). Fluorescein isothiocyanate (FITC)-conjugated rat anti-mouse P-selectin and integrin β3 antibodies were from BD Pharmingen.

### Animals

Experiments were conducted in accordance with the National Institutes of Health Guide for the Care and Use of Laboratory Animals, following approved protocols by the Institutional Animal Care and Use Committee of the University of Kentucky. The generation of a TRAF3 flox mice by homologous recombination has been described previously^39^. The TRAF3 flox line was backcrossed with C57BL/6 J (B6) mice (Jackson Laboratory) for >9 generations to generate TRAF3 flox mice on the B6 genetic background. Megakaryocyte- and platelet-specific deletion of the floxed region was then accomplished by breeding of the TRAF3 flox mice with the *Pf4*-Cre recombinase transgenic mice. Wild-type littermates, TRAF3^fl/fl^/Cre^−^ mice, were used as controls for all experiments.

### Preparation of Mouse Washed Platelets

Blood was collected from the abdominal aorta of isofluorane-anesthetized mice (8–10 weeks) using 1⁄7 volume of ACD (85 mM trisodium citrate, 83 mM dextrose, and 21 mM citric acid) as anticoagulant^40^. For each experiment, blood was pooled from three to four mice of each genotype. The platelets were then washed twice with CGS (0.12 M sodium chloride, 0.0129 M trisodium citrate, 0.03 M D-glucose, pH 6.5), and resuspended in modified Tyrode’s buffer (12 mM NaHCO_3_, 138 mM NaCl, 5.5 mM glucose, 2.9 mM KCl, 2 mM MgCl_2_, 0.42 mM NaH_2_PO_4_, 10 mM HEPES, pH 7.4) and incubated for 1 h at 22 °C before use^41^.

### Platelet Aggregation and Secretion

Platelet aggregation at 37 °C was measured by detecting changes in light transmission using a turbidimetric platelet aggregometer (Chrono-Log) with stirring (1000 rpm)^41^. ATP release was measured by adding luciferin/luciferase reagent (3~12 μl) to 250 μl of a washed platelet suspension 1 min before stimulation.

### Fibrinogen binding to platelets

Washed platelets from TRAF3^+/+^ and TRAF3^−/−^ mice (3 × 10^8^/ml) were incubated with Oregon Green-labeled fibrinogen in the presence of various doses of agonists at 22 °C for 30 min. Platelets were also incubated with Oregon Green-labeled fibrinogen in the absence of agonists at 22 °C for 30 min as a control. Fibrinogen binding to cells was analyzed by flow cytometry.

### Measurement of mouse platelet serotonin

Washed platelets (5 × 10^8^/ml, 600 μl) from TRAF3^+/+^ and TRAF3^−/−^ mice were solubilized by adding 120 μl of 6.0 M TCA. After centrifugation at 12,000 rpm for 2 min at room temperature, the TCA extract was transferred to a tube containing 1 ml of 0.05% O-phalaldehyde. Samples were boiled for 10 min, cooled on ice, and then washed twice with chloroform. Serotonin release was measured using an MDS fluorescence spectrophotometer (MDS Analytical Technologies, Sunnyvale, CA) with an excitation wavelength of 360 nm and an emission wavelength of 475 nm.

### Western blot analysis of Akt and p38 MAPK phosphorylation

Washed platelets were stimulated with thrombin or collagen in a platelet aggregometer at 37° for 2 or 5 min and then solubilized in SDS-PAGE sample buffer. Platelet or cell lysates were analyzed by SDS-PAGE on 4~15% gradient gels and immunoblotted using a rabbit monoclonal antibodies specific for the phosphorylated Akt residues Ser^473^ or phosphorylated p38 MAPK residue Thr^180^/Tyr^182^.

### Detection of TRAFs by immunoblotting

Washed platelets in Tyrode buffer (1 × 10^9^/ml) were solubilized by adding equal volume of 2% Triton X-100, 100 mM Tris, 10 mM EGTA, 0.15 M NaCl, 2 mM phenylmethylsulfonyl fluoride, and 0.2 mM E64, pH 7.4. Platelet lysates were analyzed by SDS-PAGE on 4% to 15% gradient gel and electrotransfered to polyvinylidenefluoride membranes. The presence of TRAF 2 and 3 was detected by immunoblotting with polyclonal antibodies against TRAF3^42^ or TRAF2.

### Detection of integrin and GPVI expression by flow cytometry

Washed platelets from TRAF3^+/+^ and TRAF3^−/−^ mice were incubated with FITC-conjugated rat anti-mouse β3 or GPVI monoclonal antibodies for 30 min at 22 °C and fixed by adding paraformaldehyde (1% final concentration). Integrin β3 or GPVI expression was analyzed by flow cytometry.

### Bleeding time

Seven- to 8-week-old mice were anesthetized with inhalation of 2–5% isoflurane in 100% oxygen using anesthesia equipment. The distal portion of the tail (5 mm) was amputated with a scalpel, and the tail was immersed in 0.15 M NaCl at 37 °C as previously described^43^. Time to stable cessation of the bleeding was defined as the time where no rebleeding for longer than 2 minutes was recorded. Statistical analysis was performed using nonparametric Mann-Whitney test. Genotyping of the offspring was subsequently determined by PCR analysis, using DNA extracted from tail tissue after bleeding time tests.

### *In vivo* thrombosis

An *in vivo* thrombosis model was performed as described previously^44^. Briefly, 7- to 8-week-old mice were anesthetized with intraperitoneal injection of katamine. Left carotid arteries were isolated from surrounding tissues. 21 MA-0.5PSB nanoprobe (Transonic Systems) was hooked to arteries, and blood flow was monitored with a TS420 flowmeter (Transonic Systems). After stabilization, 0.5 μL of 5.5% FeCl_3_ was applied to a filter paper disc (1-mm diameter) that was immediately placed on top of the artery for 3 minutes. After removing the filter paper, blood flow was monitored continuously until 5 minutes after occlusion. Time to occlusion was calculated as a difference in time between the removal of the filter paper and stable occlusion (no blood flow for 1 minute). Statistical analysis was performed using the nonparametric Mann-Whitney test for the evaluation of differences in median occlusion time.

## Electronic supplementary material

Supplementary data
