# Supplementary material for: TRAF3 negatively regulates platelet activation and thrombosis
Source: Sci Rep. 2017 Dec 7;7:17112. doi: 10.1038/s41598-017-17189-1 (PMC5719392; doi:10.1038/s41598-017-17189-1)
Supplement: Supplementary file 1 — Supplementary data [file 41598_2017_17189_MOESM1_ESM.pdf]

TRAF3 negatively regulates platelet activation and thrombosis

Rui Zhang[1,2], Guoying Zhang[1], Binggang Xiang, Xiaofeng Chen[3], Lijang Tang[4], Shaojun Shi[2], Yani Liu[2], Xun Ai[5], Ping Xie[6], and Zhenyu Li[1]

From [1]Division of Cardiovascular Medicine, Department of Internal Medicine, College of Medicine, University of Kentucky, 741 South Limestone Street, Lexington, KY 40536, USA, [2]Department of Pharmacy, Union Hospital, Tongji Medical College, Huazhong University of Science and Technology, 1277 Jiefang Avenue, Wuhan, P.R. China . [3]Taizhou Hospital, Wenzhou Medical University, Taizhou, 317000, China, [4]Department of Cardiology, Zhejiang Hospital, Hanzhou, 310013, China, [5]Department of Physiology and Biophysics, Rush University, 1750 W Harrison St, Chicago, IL60612, USA, [6]Department of Cell Biology and Neuroscience, Rutgers University, 604 Allison Road, Piscataway, NJ 08854, USA

Address correspondence to: Zhenyu Li, Department of Internal Medicine, College of Medicine, University of Kentucky, 741 South Limestone Street, Lexington, KY 40536. Tel. 859-257 0528; E-mail: zhenyuli08@uky.edu.

Supplementary Figure S1. Fibrinogen binding in response to ADP and CRP in TRAF3$^{+/+}$ and TRAF3$^{-/-}$ platelets. (a and b) Washed platelets from TRAF3$^{+/+}$ and TRAF3$^{-/-}$ mice (3 x 10^8/ml) were incubated with Oregon Green-labeled fibrinogen in the presence of various concentrations of ADP (a) or CRP (b) at 22°C for 30 min. Platelets were also incubated with Oregon Green-labeled fibrinogen in the absence of thrombin at 22°C for 30 min as a control. Fibrinogen binding to platelets was analyzed by flow cytometry (n=3).

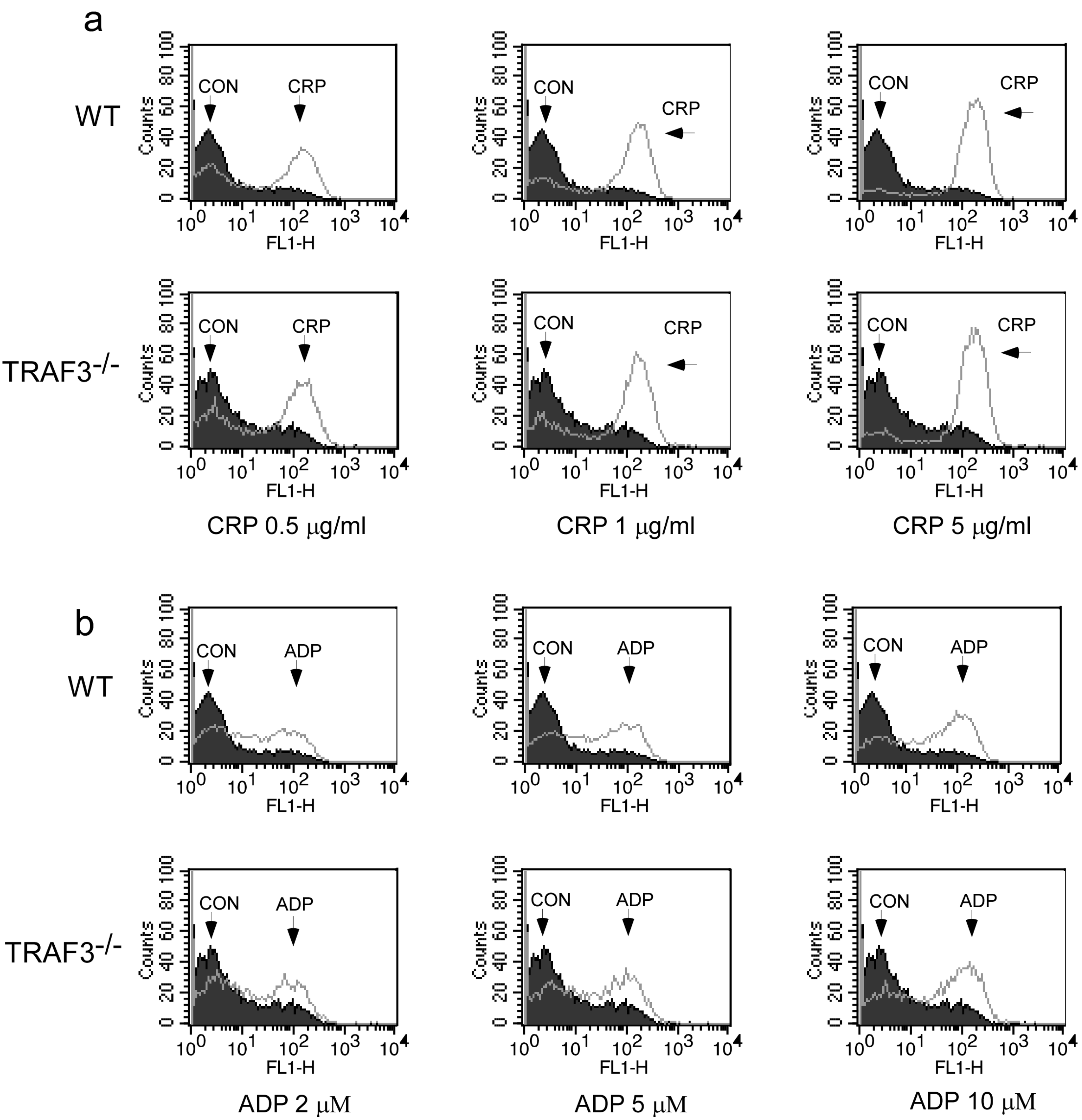
a
WT
TRAF3-/-
CON
CRP
Counts
FL1-H
CRP 0.5 μg/ml
CRP 1 μg/ml
CRP 5 μg/ml
b
WT
TRAF3-/-
CON
ADP
ADP 2 μM
ADP 5 μM
ADP 10 μM

Supplementary Figure S2. Expression of integrin, GPVI, CD40 and TRAFs were not affected in TRAF3$^{-/-}$ mice. (a) Washed platelets from TRAF3$^{+/+}$ and TRAF3$^{-/-}$ mice were incubated with a FITC-labeled rat anti-mouse FITC-labeled control IgG or an anti-β3 monoclonal antibody (BD Pharmagen) for 30 min at 22°C and analyzed by flow cytometry. Data shown are representative of three independent experiments. (b) Washed platelets from TRAF3$^{+/+}$ and TRAF3$^{-/-}$ mice were incubated with a FITC-labeled rat anti-mouse GPVI monoclonal antibody (Enfret Analytics) or FITC-labeled control IgG for 30 min at 22°C and analyzed by flow cytometry. Data shown are representative of three independent experiments. (c) Detection of GPVI and integrin β3 in platelet lysates by Western blot. (d) Statistical data of densitometric analysis of GPVI, integrin β3 and TRAF2 (mean ± SD; n=4). (e) Detection of TRAF2 (NeoBiolab Inc) in platelet lysates by Western blot. (f) Detection of TRAF5 (Cell Signaling Technology), 6 (Cell Signaling Technology), 7 (Novus Biologicals), and CD40 (Santa Cruz Biotechnology) in platelet lysates by Western blot. Shown was a representative of four independent experiments.

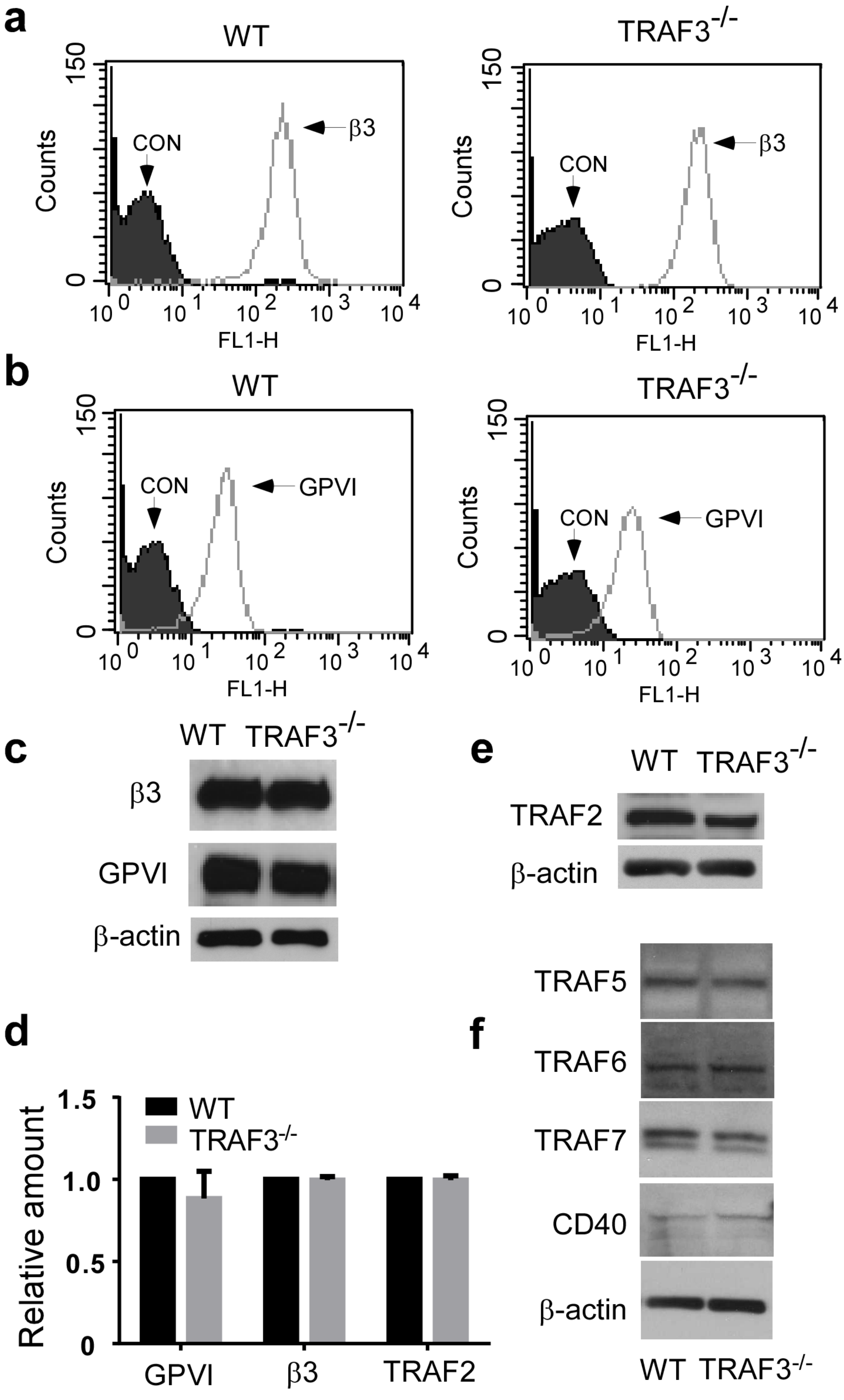

Supplemental Figure 2
